# Genetic Characterization and Passage Instability of a Hybrid Plasmid Co-Harboring *bla*_IMP-4_ and *bla*_NDM-1_ Reveal the Contribution of Insertion Sequences During Plasmid Formation and Evolution

**DOI:** 10.1128/Spectrum.01577-21

**Published:** 2021-12-15

**Authors:** Xi Li, Jintao He, Yan Jiang, Minfei Peng, Yunsong Yu, Ying Fu

**Affiliations:** a Centre of Laboratory Medicine, Zhejiang Provincial People’s Hospital, People's Hospital of Hangzhou Medical College, Hangzhou, Zhejiang Province, China; b Department of Infectious Diseases, Sir Run Run Shaw Hospital, School of Medicine, Zhejiang University, Hangzhou, Zhejiang Province, China; c Key Laboratory of Microbial Technology and Bioinformatics of Zhejiang Province, Hangzhou, Zhejiang Province, China; d Regional Medical Center for National Institute of Respiratory Diseases, Sir Run Run Shaw Hospital, Zhejiang University School of Medicine, Hangzhou, China; e Zhejiang Chinese Medical University, Hangzhou, Zhejiang Province, China; f Department of Clinical Laboratory, Taizhou Hospital of Zhejiang Province, Linhai, Zhejiang Province, China; g Department of Clinical Laboratory, Sir Run Run Shaw Hospital, School of Medicine, Zhejiang University, Hangzhou, Zhejiang Province, China; h Key Laboratory of Precision Medicine in Diagnosis and Monitoring Research of Zhejiang Province, Hangzhou, Zhejiang Province, China; Dublin City University

**Keywords:** carbapenem resistance, *Enterobacterales*, *bla*_IMP-4_, *bla*_NDM-1_, genetic characterization, instability, plasmid formation, evolution

## Abstract

Carbapenemase is the predominant enzyme in the mechanism leading to *Enterobacterales* resistance to carbapenems, but only a limited number of isolates harbor double classes/types of carbapenemase. Here, an IMP-4 and NDM-1 producer named Klebsiella michiganensis 7525 is reported, and the co-harboring plasmid is further characterized. K. michiganensis 7525 was positive for the *bla*_IMP-4_ and *bla*_NDM-1_ genes by the NG-Test Carba-5 method and PCR followed by sequencing, and both were located on the same plasmid (designated pKOX7525_1) according to S1-PFGE with Southern blot experiments. pKOX7525_1 was capable of transconjugation with an efficiency of 4.3 × 10^−8^ in a filter mating experiment. Whole-genome sequencing and bioinformatics analysis confirmed that the plasmid was novel, clustered to the incompatibility type of IncHIB/IncFIA/IncR and presented high similarity to a *bla*_IMP-4_-carrying IncHIB plasmid (pA) published with 79% coverage and 100% sequence identify. In contrast, a large-fragment insertion and inversion mediated by IS*26* was observed on the plasmid, which introduced a genetic hybrid zone with multiple resistance genes, including *bla*_NDM-1_, to the plasmid. In the transconjugants, the presence of pKOX7525_1 had a negative impact on bacterial fitness. *In vitro* evolution experiments showed that pKOX7525_1 in the transconjugant could not be stably inherited after 10 days of passage and that *bla*_NDM-1_ could be lost during repeated laboratory passage. Our study not only reports a novel plasmid co-harboring *bla*_IMP-4_ and *bla*_NDM-1_ but also highlights the putative pathway of plasmid formation and evolution by means of genetic rearrangement through sequence insertion and homologue recombination, which may have critical value for plasmid research and increase awareness of carbapenem-resistant *Enterobacteriaceae* (CRE).

**IMPORTANCE** In this study, we characterized a novel plasmid from a carbapenem-resistant K. michiganensis (CRKM) isolate, which harbors two metallo-β-lactamases (MBLs), IMP-4 and NDM-1, is capable of transconjugation and contains three replicons. Our results first expand the diversity of plasmids co-harboring carbapenemase genes in *Enterobacterales*, which exhibits epidemic importance in bacterial resistance. Additionally, we investigated the origin and formation of this MBL double-positive plasmid based on comparative genomics analysis, which indicated that IS*26* plays a vital role through continuous genetic rearrangements. Moreover, this plasmid is unstable in transconjugants during passage at the multidrug-resistant (MDR) region of *bla*_NDM-1_, with fluctuating stability under varying antibiotic selection, highlighting auspicious considerations regarding recognition of the complexity and plasticity of plasmids in evolution and re-emphasizing clinical infection control inspired by CRE.

## INTRODUCTION

Infectious diseases caused by carbapenem-resistant *Enterobacterales* (CRE), which are always associated with significantly high morbidity and mortality, have been an urgent and continuous threat to public health throughout the world (1). The prevalence of carbapenem resistance in *Enterobacterales* is mainly mediated by the rapidly increasing prevalence of carbapenemase gene carriage. Carbapenemase production, such as KPC-2, NDM-1, IMP-4, VIM-1, and OXA-48, is the primary mechanism by which CRE isolates present resistance to carbapenem agents, which alone have strong hydrolysis activity against carbapenems when fully expressed, and carrier isolates with a single copy gene have high MIC values for all β-lactams (2, 3). Unfortunately, higher-level carbapenem resistance can occur when these enzymes are found in combination with other β-lactamases, when multiple carbapenemases coexist, or with porin changes leading to permeability defects, which may substantially escalate the global public-health threat of the “superbug” (4, 5).

With the wide application of high-throughput sequencing on clinical isolates, an increasing number of CREs have been completely sequenced by whole-genome sequencing (WGS). However, most of their sequences have affirmed that the primary form of carbapenem-resistant genes in one isolate is one type or one copy, while very few strains possess multiple types simultaneously, and CRE with coexistence of the same type of carbapenemase genes on the same plasmid has rarely been reported. Here, we characterized carbapenem-resistant Klebsiella michiganensis (CRKM), which co-harbors *bla*_IMP-4_ and *bla*_NDM-1_ on the same plasmid, and explored the phenotypic and genotypic characteristics of the plasmid. We also studied dynamic changes in the *bla*_IMP-4_ and *bla*_NDM-1_-coharbouring plasmid following conjugation experimental evolution.

## RESULTS AND DISCUSSION

### Phenotypic and antibiotic resistance genes characteristics of *K. michiganensis* stain 7525.

Previous studies have showed Klebsiella spp. is the main genus producing double or triple carbapenemases, and the predominant coexistence forms of carbapenemase types within the same strain include KPC with NDM or KPC with IMP (6). Meanwhile, the coding genes were mostly located on different plasmids in the same strain and presented varying plasmid transferability to recipient cells by conjugation (5). The research published by Gao et al. had epidemic importance because it identified Klebsiella pneumoniae isolates co-producing KPC-2 and NDM-1 in diverse provinces in China, which highlights a potential threat to global public health (5, 7).

**FIG 1:**
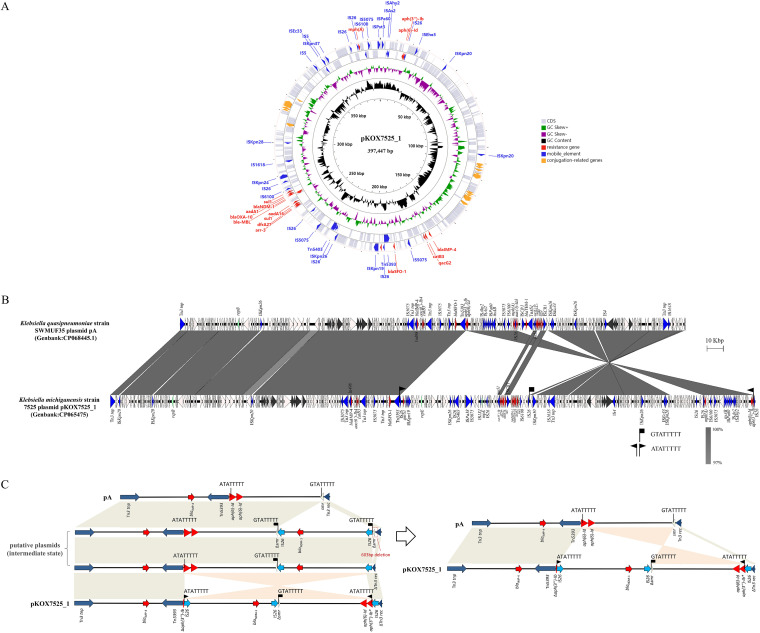
Characterization of *bla*_IMP-4_ and *bla*_NDM-1-_coharboring plasmid pKOX7525_1. (A) Plasmid structure of pKOX7525_1. (B) Schematic illustration comparing the structural features of plasmid pKOX7525_1 with sequences of plasmid pA (CP068445). Gray shading and squares indicate homologies between the corresponding genetic loci on each plasmid. Arrows indicate open reading frames, with arrowheads indicating the direction of transcription: red, antibiotic resistance-encoding genes; black, individual conjugation-related genes; blue, transposon- and integron-associated genes; green, replication-associated genes; other genes are shown by white arrows. (C) The possible formation route from pA to pKOX7525_1.

As a member of Klebsiella genus, K. michiganensis is recognized as a significant pathogen associated with nosocomial infections since it was first reported to have been isolated from a toothbrush holder in 2013 (8). Carbapenem-resistant K. michiganensis has circulated in many countries in recent years, and strains co-producing NDM and other carbapenemases have been reported (9–11). K. michiganensis strain 7525 was first identified as Klebsiella oxytoca by MALDI-TOF MS and then revised to K. michiganensis by using the average nucleotide identity (ANI) calculator. The ANI value between K. michiganensis strain 7525 and K. oxytoca (CP027426.1) is 92.10%, much lower than 97.82% of our strain and K. michiganensis (AP022547.1). This strain was resistant to β-lactams, including carbapenems (meropenem and imipenem), quinolones (ciprofloxacin and levofloxacin), aztreonam, piperacillin-tazobactam, and ceftazidime-avibactam, whereas it was susceptible to amikacin, tigecycline, and colistin (Table 1). Interestingly, the *bla*_IMP-4_ gene and *bla*_NDM-1_ gene were detected, both of which were located on the same plasmid with a size of approximately 398 kb (Fig. S1). In addition, multiple antibiotic resistance genes (ARGs) were also annotated on the same plasmid (named pKOX7525_1), and *bla*_OXY-5_ and *bla*_CTX-M-15_ were located on the chromosome (Table S2, Fig. 1A).

**TABLE 1:** Phenotypic and genotypic characteristics of strains used in our study*^a^*

Name	Phenotype of AST (MICs, μg/mL)												Genotype of carbapenem-resistance
	CAZ	FEP	TZP^*b*^	CZA	IMP	MEM	ATM^*b*^	CIP	LEV^*b*^	AMK	TGC	COL	
K. michiganensis strain 7525	>128	>128	>256	>128	>128	64	>256	64	16	2	0.125	0.25	*bla*_IMP-4_, *bla*_NDM-1_
E. coli J53 (pKOX7525_1)	>128	>128	>256	>128	>128	64	6	2	1	2	0.125	0.25	*bla*_IMP-4_, *bla*_NDM-1_
XH2065	>128	>128	>256	>128	>128	64	4	2	0.5	4	0.125	0.25	*bla*_IMP-4_, *bla*_NDM-1_
XH2066	>128	>128	4	128	32	8	8	4	0.5	8	0.125	0.25	*bla*_IMP-4_
XH2067	>128	>128	4	128	32	16	6	2	0.5	2	0.125	0.25	*bla*_IMP-4_
XH2068	≤0.125	≤0.125	0.5	0.125	0.5	≤0.125	0.094	≤0.125	0.016	2	0.125	0.25	ND
E. coli J53	≤0.125	≤0.125	0.5	0.125	0.5	≤0.125	0.125	≤0.125	0.016	2	0.125	0.25	ND

a CAZ, ceftazidime; FEP, cefepime; TZP, piperacillin-tazobactam; CZA, ceftazidime-avibactam; IMP, imipenem; MEM, meropenem; ATM, aztreonam; CIP, ciprofloxacin; LEV, levofloxacin; AMK, amikacin; TGC, tigecycline; COL, colistin.

b MIC values were determined by Etest. ND, not detected.

In China recently, ongoing studies have completed WGS of double- or multiple-carbapenemase-producing CREs and have performed further bioinformatic analyses (Table S3). However, among plasmids in the NCBI nucleotide database, very few sequences were positive for both *bla*_NDM-1_ and *bla*_IMP-4_. Wang et al. first reported a K. oxytoca strain simultaneously producing NDM-1, IMP-4, and KPC-2 carbapenemases, which were located on three plasmids. The Inc types of pKOX3-p3-NDM, pKOX3-P4-IMP, and pKOX3-P5-KPC in their study were Inc*X3*, Inc*N*, and Inc*P-6*, respectively, all of which belonged to epidemic plasmids in China (12). One possible mechanism producing double- or multiple-carbapenemase is mediated by direct transmission of epidemic plasmids. Another plasmid (IncW-type) from Providencia rettgeri in the NCBI nucleotide database co-harbors the *bla*_IMP-4_ and *bla*_NDM-1_ genes (GenBank accession no. MH882484) and is only 41,938 bp in length, showing a significant difference from our sequence. Under epidemic conditions of genotypes of carbapenem resistance, the emergence of IMP-4 and NDM-1 co-producing CRKM updates our understanding of the distributions of resistance genes.

### Proposed formation mechanism of pKOX7525_1 mediated by IS*26*.

pKOX7525_1 is 397,447 bp long with a 49.1% GC content, and 541 predicted open reading frames (ORFs) (Table S2). Three replicons were identified, which produced a novel plasmid clustered into the incompatibility groups of IncHI5B, IncFIA and IncR. In addition, it has been heavily modified by mobile elements, including complete insertion sequences (ISs) or incomplete elements of at least 40 copies (Fig. 1). The determined class I integron In*498* (*bla*_IMP-4_-*itrA-qacG2-aacA4-catB3* cassette array) is commonly associated with a low level of carbapenem resistance in China and conserved among the *bla*_IMP-4_ plasmids in *Enterobacterales*, such as pA708-1 from Klebsiella quasipneumoniae (GenBank accession no. CP026369.1), pA708-IMP from K. pneumoniae (GenBank accession no. MF344567.1) and unnamed plasmids from Klebsiella aerogenes (GenBank accession no. CP028952.1).

pKOX7525_1 shows 100% nucleotide sequence identity and 79% sequence coverage with the pA plasmid (GenBank accession no. CP068445), which is an IncHI5B plasmid recovered from K. quasipneumoniae strain SWMUF35 (Fig. 1B). However, a short divergent region carrying multiple ARGs was detected. Further comparison between the two plasmids indicated that pKOX7525_1 may originate from the pA plasmid, and several intermediate states may occur.

Mobile genetic elements, particularly ISs, are capable of reorganizing sequences in plasmids, and among all ISs, IS*26* seems to play a major role in the rapid dissemination of antibiotic resistance genes in Gram-negative bacteria (13–15). The insertion of one or more IS*26* elements mediated several insertion events involving a plethora of ARGs, including the *bla*_NDM-1_-carrying multidrug-resistant (MDR) region. IS*26* copies also lead to DNA sequence inversion in pKOX7525_1 via intramolecular replicative transposition in *trans*. In our study, pKOX7525_1 resulted from complex IS*26*-mediated formation that led to two major differences compared with the genome of pA: an inverted configuration and an inserted *bla*_NDM-1_-bearing MDR region. Sequence alignment between pA and pKOX7525_1 identified a 78.1-kb redundant MDR fragment bracketed by IS*26* located in the same orientation, but the flanked 8-bp site sequences (ATATTTTT; GTATTTTT) were not the same. In addition, two copies of IS*26* were adjacent to the 211.8-kb inverted configuration in pKOX7525_1 and showed opposite orientations, which were flanked by an 8-bp target site duplication (TSD) ATATTTT. The left side of the inverted configuration and inserted MDR region were flanked by the same IS*26* (Fig. 1C). Outside of the three IS*26* copies of pKOX7525_1, a 16-bp truncated *aph(*6*)-Id* gene was adjacent to the left IS*26*, a truncated *smr* gene encoding an interrupted multidrug efflux SMR transporter was adjacent to the middle IS*26*, and a truncated *rec* gene encoding an interrupted recombinase family protein was adjacent to the right IS*26*. The two genes (*smr* and *rec*) were adjacent in plasmid pA, and an 8-bp target site GTATTTTT was found in the *smr* gene, indicating that the MDR region had been inserted into the multidrug efflux SMR transporter encoding gene *smr*. The target site ATATTTT was found in the ARG *aph(*6*)-Id*, and this inversion event led to changes in the first four amino acids of the *aph(*6*)-Id* gene, resulting in the formation of a novel *aph(*6*)-Id* variant. Considering the characteristics of the transposition of IS*26* (13) and the target site GTATTTTT, which was found around only one IS*26*, IS*26* most likely first mediated the insertion of the 78.1-kb MDR region and then mediated the inversion of a 211.8-kb fragment including the MDR region (Fig. 1C), indicating that IS*26* is involved in the accumulation of resistance genes and the rearrangement of multidrug resistance regions.

### Genetic structures of the *bla*_NDM-1_-bearing inserted MDR region.

**FIG 2:**
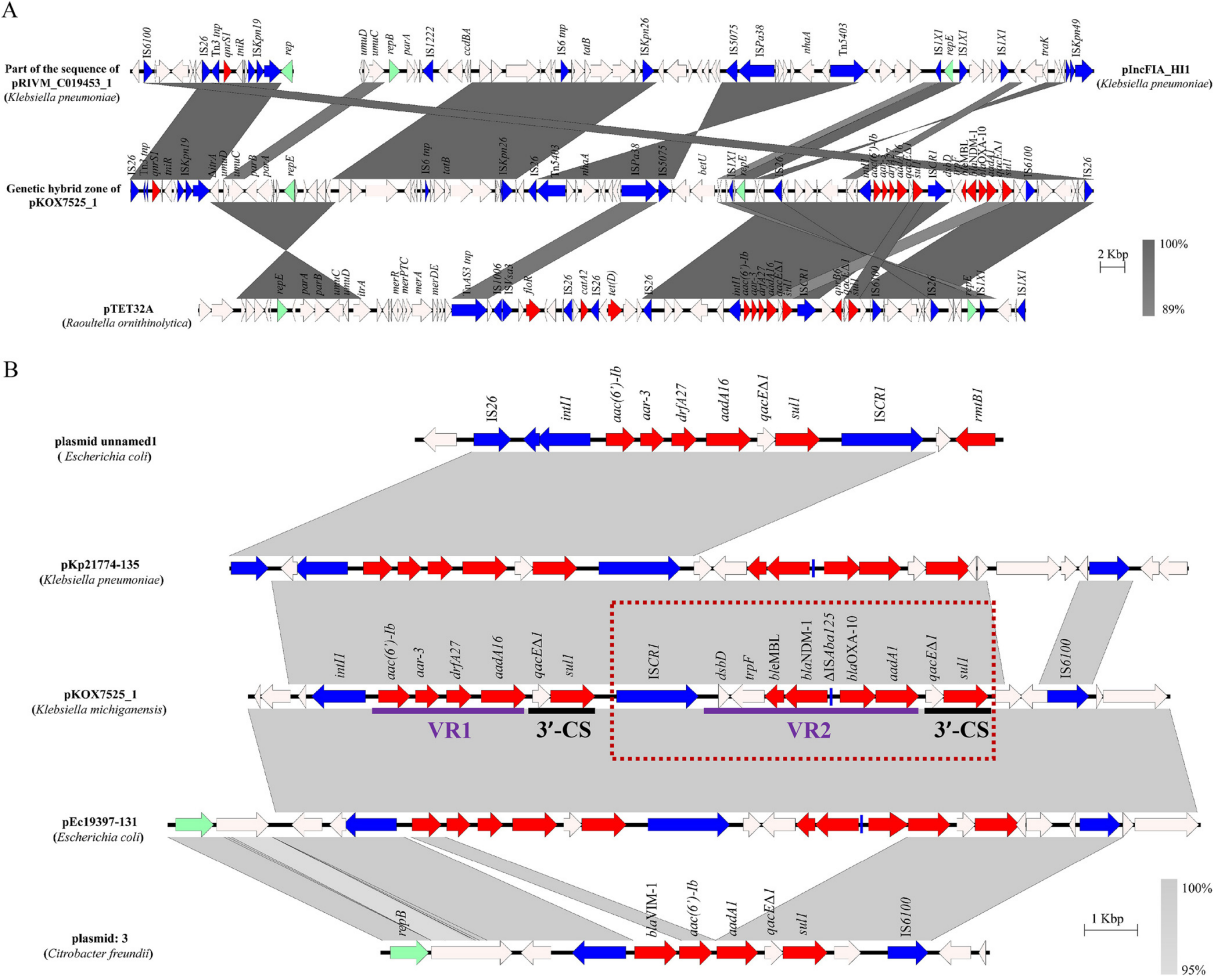
Genomic analyses of the inserted *bla*_NDM-1_-carrying MDR region. (A) Scaled, linear diagrams comparing the sequences between the inserted *bla*_NDM-1_-carrying MDR region, pIncFIA_HI1 (CP060424), pTET32A (CP067414), and the part of the sequence of pRIVM_C019453_1 (CP068909). (B) Genetic context of *bla*_NDM-1_ on pKOX7525_1 and related sequences of plasmids. The GenBank accession numbers are CP020510, MG878868, MG878866, and LS992177. Gray shading and squares indicate homologies between the corresponding genetic loci on each plasmid. Arrows indicate open reading frames, with arrowheads indicating the direction of transcription: red, antibiotic-resistant encoding genes; blue, transposon- and integron-associated genes; green, replication-associated genes; Other genes are shown as white arrows.

The *bla*_NDM-1_-bearing MDR region of pKOX7525_1, which is labeled as the genetic hybrid zone (GHZ) in Fig. 2A, was 78,115 bp in length and contained IncFIA and IncR replicons, indicating a plasmid origin. In addition to the carbapenemase gene *bla*_NDM-1_, this putative plasmid region also harbored multiple ARGs, as shown in Fig. 2A. However, no plasmid was similar to this MDR region in sequence, and the highest query coverage (GenBank accession no. CP021168) was merely 56% in the NCBI database. The sequence of the MDR region had 99.9% nucleotide identity at 52% coverage to pTET32A (GenBank accession no. CP067414) from Raoultella ornithinolytica strain TET32, which also comprised the IncFIA and IncR replicons, indicating that it might have a common origin with pTET32A, while the remaining sequence being unmatched implies that it might originate from other plasmids, such as pIncFIA_HI1 (GenBank accession no. CP060424) and pRIVM_C019453_1 (GenBank accession no. CP068909). Interestingly, the sequences upstream and downstream of the MDR region were continuous on pRIVM_C019453_1, suggesting that this putative plasmid region from the plasmid state to the MDR region may be separated from IS*26* in the middle of the sequence, as in pRIVM_C019453_1 (Fig. 2A).

The inserted MDR region of pKOX7525_1 harbored two ARG cassette arrays. The class I integron was organized as a 5′-conserved segment (5′-CS) (*intI1*), variable region 1 (VR1) (*aac(6′)-Ib*- *arr-3*-*dfrA27-aadA16*), and 3′-CS (*qacE*Δ*1*/*sul1*). The carbapenemase gene *bla*_NDM-1_ was embedded in the IS*CR1* element consisting of IS*CR1*, VR2 (*dsbD*-*traF*-*ble*_MBL_-*bla*_NDM-1_-ΔIS*Aba125*-*bla*_OXA-10_- *aadA1*), and 3′-CS (*qacEΔ1*/*sul1*) (Fig. 2B), which can mobilize the *bla*_NDM-1_ gene to move into the 3′-CS by rolling-circle transposition (16). These cassette arrays have also been found in diverse isolates with different genetic contexts, including Escherichia coli plasmids pEc19397–131 (GenBank accession no. MG878866) and pEc21617–310 (GenBank accession no. MG878867), Citrobacter werkmanii plasmid pCB1_SE1_NDM (GenBank accession no. MK124610), Enterobacter hormaechei plasmid pEH_316-3 (GenBank accession no. CP078058), and K. pneumoniae plasmids pLK78 (GenBank accession no. KJ440075) and pKp21774-135 (GenBank accession no. MG878868), suggesting the worldwide dissemination potential of the *bla*_NDM-1_ gene. IS*CR*s are a class of mobile elements that have been implicated in capturing genes or gene fragments and are often found in the vicinity of *bla*_NDM-1_, which is also involved in the mobilization of *bla*_NDM-1_ (17–19). The IS*CR* element is responsible for the mobilization of *bla*_NDM-1_ by rolling-circle replication and can be lost after experimental passage under nonselective conditions (16). In our study, we also observed that the *bla*_NDM-1_ gene was lost despite retaining the plasmid after 5 days of passage in an antibiotic-free environment. After genome alignment to a reference sequence (pKOX7525_1), our sequence analysis showed that an obvious coverage deletion was observed in the coverage map of XH2067-pKOX7525_1 or XH2069-pKOX7525_1, and corresponds to the IS*CR1* element (IS*CR1*-*dsbD*-*traF*-*ble*_MBL_-*bla*_NDM-1_-ΔIS*Aba125*-*bla*_OXA-10_-*aadA1*-*qacEΔ-sul1*) (Fig. S2). Therefore, the IS*CR1* element may move into the 3′-CS (*qacEΔ1*/*sul1*) of a class 1 integron (*intI1*-*aac(6′)-Ib*- *arr-3*-*dfrA27-aadA16*-*qacE*Δ*1*-*sul1*) by homologous recombination, indicating that pKOX7525_1 obtained *bla*_NDM-1_ by IS*CR1*-mediated homologous recombination and further illustrating the possible mechanism of pKOX7525_1 formation.

### The biological feature of pKOX7525_1.

**FIG 3:**
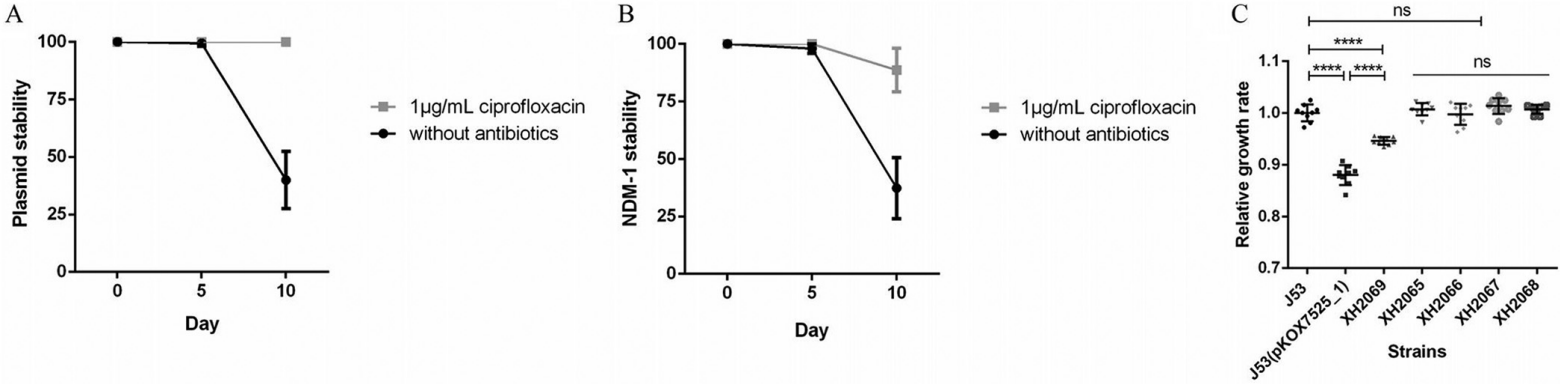
Characterization of the biological features of pKOX7525_1. Line charts of (A) plasmid stability and (B) NDM-1 stability of pKOX7525_1 of the transconjugant E. coli J53 (pKOX7525_1) in LB broth without or with antibiotics. (C) Growth rates of the recipient strain J53, the transconjugant E. coli J53 (pKOX7525_1) and five isolates of transconjugants after experimental passage. The experiment was repeated in triplicate. Representative results of three independent experiments are shown, and the data are the mean standard deviation (SD). ****, *P* < 0.0001 (one-way ANOVA).

Conjugation assays showed that pKOX7525_1 can be successfully transferred to an E. coli J53 recipient strain with a low efficiency of 4.3 × 10^−8^ transconjugants per recipient cell, and the carbapenem-resistant phenotype of the corresponding transconjugant E. coli J53 (pKOX7525_1) could be fully expressed and caused a sharp increase in the MIC toward carbapenems, cephalosporins, and cephalosporin/β-lactamase inhibitors (Table 1). To estimate the plasmid stability and *bla*_NDM-1_ stability of pKOX7525_1, we performed experimental passage experiments with the transconjugant E. coli J53 (pKOX7525_1) under both antibiotic and antibiotic-free conditions. According to the results of antimicrobial susceptibility testing (AST) (Table 1) and characteristics of IS*CR1* elements (16), we chose ciprofloxacin as the selective antibiotic. pKOX7525_1 remained stable (stability = 99.3%) for 5 days of passage in an antibiotic-free environment, but the stability decreased significantly (stability = 40%) after 10 days of passage (Fig. 3A), indicating that the plasmid could not be stably inherited, possibly due to the large size of the plasmid. The loss of *bla*_NDM-1_ was also observed at a low frequency (11.3% of the colonies) in the antibiotic environment where plasmid stability was 100% for E. coli J53 (pKOX7525_1) (Fig. 3A and B). The *bla*_NDM-1_ stability of E. coli J53 (pKOX7525_1) in an antibiotic-free environment (the plasmid stability data are included) was 98% (day 5) and 37.3% (day 10) (Fig. 3B). To identify the effects of *bla*_NDM-1_ loss in pKOX7525_1 on the host, we selected some isolated experimental passages for AST and growth rate determination (Table 1, Table S1). The AST results showed that XH2066 and XH2067, two transconjugants containing *bla*_IMP-4_ but with *bla*_NDM-1_ loss, displayed decreased MICs toward carbapenems (imipenem and meropenem) and cephalosporin/β-lactamase inhibitors (ceftazidime-avibactam and piperacillin-tazobactam) compared with that of the parental strain E. coli J53 (pKOX7525_1) or XH2065 (a transconjugant containing *bla*_IMP-4_ and *bla*_NDM-1_) but were still significantly higher than that of E. coli J53 or XH2068 (a transconjugant with pKOX7525_1 loss) (Table 1), indicating that the high level of carbapenem resistance induced by pKOX7525_1 is caused by the synergistic effect of IMP-4 and NDM-1. The growth rate results showed that the transconjugant exhibited a significantly decreased growth rate compared to the recipient strain E. coli J53 (Fig. 3C), indicating that pKOX7525_1 confers a fitness cost on the host. Interestingly, we observed that all the strains after 10 days of passage (XH2065, XH2066, XH2067, and XH2068) had similar growth rates to E. coli J53, but these rates were much higher than that of the parental strain E. coli J53 (pKOX7525_1) with or without pKOX7525_1 or *bla*_NDM-1_. In addition, the growth rate of the strains after 5 days of passage (XH2069) was between those of XH2067 and the parental strain E. coli J53 (pKOX7525_1), suggesting that the host had gradually developed compensatory mechanisms associated with pKOX7525_1 carriage during the passage process, which may help the host better carry pKOX7525_1. The detailed compensatory mechanisms require further experimental confirmation.

## CONCLUSION

Here, we characterized a novel plasmid from a CRKM isolate named pKOX7525_1, which harbors two metallo-β-lactamases, IMP-4 and NDM-1, is capable of transconjugation and contains three replicons. Our results first expand the diversity of plasmids co-harboring carbapenemase genes in *Enterobacterales*, which exhibits epidemic importance in bacterial resistance. Additionally, we investigated the origin and formation of this MBL double-positive plasmid based on comparative genomics analysis, which indicated that IS*26* plays a vital role through continuous genetic rearrangements. Moreover, pKOX7525_1 is unstable in transconjugants during passage at the MDR region of *bla*_NDM-1_, with fluctuating stability under varying antibiotic selection, highlighting auspicious considerations regarding recognition of the complexity and plasticity of plasmids in evolution and re-emphasizing clinical infection control inspired by CRE.

## MATERIALS AND METHODS

### Strain identification and antimicrobial susceptibility testing.

In August 2020, a 49-year-old patient diagnosed with B-cell acute lymphoblastic leukemia was admitted to a grade three hospital in Zhejiang Province, China, for allogeneic haematopoietic cell transplantation (allo-HSCT). She developed a urinary tract infection immediately after she received treatment with intensified myeloablative conditioning regimens. A MDR isolate (K. michiganensis stain 7525) was recovered from urinary specimens, which was identified by matrix-assisted laser desorption/ionization time-of-flight (MALDI-TOF) mass spectrometry (MS) (bioMérieux, France). Both dilution methods combined with E-tests (bioMérieux, France) were then used for phenotyping and AST confirmation according to the standard protocol of the Clinical and Laboratory Standards Institute (CLSI) (20). The MIC breakpoints of CLSI M100 30th Edition were used for interpretation of the AST results (20), except for tigecycline and colistin, which were interpreted by the criteria of the European Committee on Antimicrobial Susceptibility Testing (EUCAST) for *Enterobacterales*.

### Confirmatory experiments of carbapenem-resistant genotype.

The NG-Test Carba 5 *in vitro* multiplex immunoassay was used for carbapenemase detection, and PCR amplification and sequencing were performed on carbapenem-resistant genes (21, 22). Pulsed-field gel electrophoresis (PFGE) after S1 restriction enzyme digestion was used to produce the fingerprint of the plasmid, and then southern blotting with a specific probe was applied to obtain the localization of carbapenem-resistant genes according to a previous report (23).

### Whole-genome sequencing and genomic analysis.

Genomic DNA extraction was performed with a QIAamp DNA minikit (Qiagen Valencia, CA). An Illumina HiSeq X 10 platform (Illumina, San Diego, CA) and a MinION device (Oxford Nanopore Technologies Inc., UK) were used for WGS. Unicycler v0.4.8 (24) and Prokka 1.11 (25) were used to generate the complete genome sequence and for gene annotation, respectively. The online pubmlst tool was used for to detect multilocus sequence typing (MLST). Plasmid replicon types of plasmids were determined using the PlasmidFinder and KpVR tools (26). ARGs were identified using the ResFinder database with Abricate 0.8 and BacAnt (27). A graphic map was generated by the CGView server. ISFinder and oriTfinder were used for data analysis. Sequence comparisons were performed using BLASTn v2.4.0 (28) and visualized using Easyfig v2.2.3 (29).

### Filter mating experiment.

A filter mating experiment was performed to investigate the transfer ability of the plasmid using Escherichia coli J53 as a recipient, as previously reported (30). Conjugation efficiency was calculated by dividing the number of transconjugants (CFU/mL) by the number of donor cells (CFU/mL). If no colonies grew on a double selection plate when 50 μl of the mixture was applied, the limit of detection (<20 CFU/mL) was taken, and a minimal value of 19 was used as the number of transconjugants for further calculation. ASTs were then tested on transconjugants positive for carbapenemase genes.

### Stability of the plasmid and *bla*_NDM-1_.

To determine the stability of the plasmid and *bla*_NDM-1_ in the transconjugant, a passage experiment was completed under conditions with and without antibiotics. Briefly, three single colonies of E. coli J53 (pKOX7525_1) were inoculated in 2 mL Luria-Bertani (LB) broth (Sangon Biotech, Shanghai, China) with 1 μg/mL ciprofloxacin or without antibiotic under shaking (200 rpm) at 37°C. Two microlitres of overnight culture was collected and used for inoculation at a 1:1,000 dilution every day. All evolved lineages were passaged daily for a total of 10 days, and the cultures were serially diluted using PBS and grown on Mueller-Hinton (MH) agar (Oxoid, Hampshire, UK) with no antibiotics every 5 days. Then, 50 clones were chosen and transferred to MH plates supplemented with 32 μg/mL ceftazidime for selection. Biological triplicates and technical triplicates were completed in this experiment.

To calculate the *bla*_NDM-1_ stability, *bla*_IMP-4_ and the *bla*_NDM-1_ in clones grown on antibiotic-supplemented agar as the marker sequences of the plasmid and the IS*CR1* element were determined by PCR with the primers IMP-4-forward (5′-TTGACACTCCATTTACGGCT-3′′), IMP-4-reverse (5′-AACCGCCTGCTCTAATGTAA-3′′), NDM-1-forward (5′-TTGCCCAATATTATGCACCC-3′′) and NDM-1-reverse (5′- GCCGGGGTAAAATACCTTGA-3′′). Plasmid stability was calculated as the number of cells observed on an MH agar plate containing 32 μg/mL ceftazidime versus cells observed on an MH agar plate without antibiotics.

### Growth rate determination.

To investigate the fitness cost of transconjugants in an antibiotic-free environment, growth rate determination was performed as previously described (16). The growth curves were estimated by GraphPad Prism version 6 using one-way analysis of variance (ANOVA) followed by Tukey’s multiple comparisons tests. The values returning an adjusted *P value* of 0.05 were considered as significant.

### Data availability.

Sequences were submitted to National Center for Biotechnology Information (NCBI) with accession numbers of CP065474 (chromosome of *K. michiganensis* strain 7525) and CP065475 (pKOX7525_1), respectively.
