# Supplementary material for: Genetic Characterization and Passage Instability of a Hybrid Plasmid Co-Harboring blaIMP-4 and blaNDM-1 Reveal the Contribution of Insertion Sequences During Plasmid Formation and Evolution
Source: Microbiol Spectr. 2021 Dec 15;9(3):e01577-21. doi: 10.1128/Spectrum.01577-21 (PMC8672901; doi:10.1128/Spectrum.01577-21)
Supplement: SUPPLEMENTAL FILE 1 — Supplemental material. Download SPECTRUM01577-21_Supp_1_seq8.pdf, PDF file, 0.8 MB [file spectrum01577-21_supp_1_seq8.pdf]

Supplementary Materials

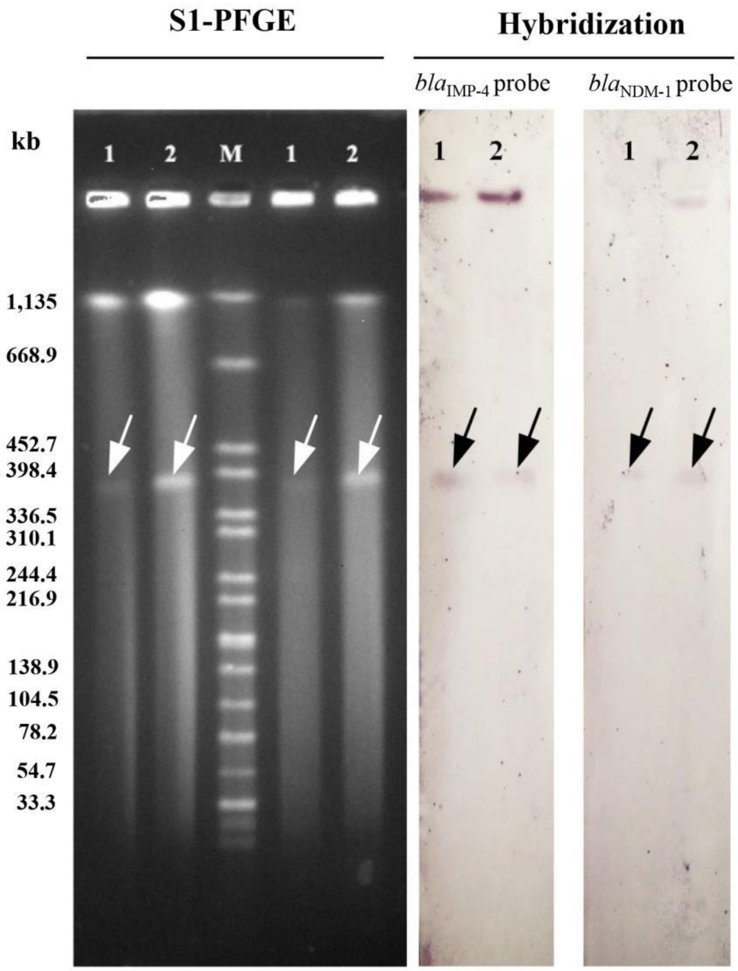

Supplementary Figure 1. S1-PFGE and Southern blot hybridization

1, *K. michiganesis* strain 7525; 2, transconjugant *E. coli* J53 (pKOX7525_1); M, *Salmonella enterica*, serotype Braenderup H9812 digested by XbaI restriction enzyme. The black arrows are the positive signals via southern blot hybridization with *bla*$_{IMP-4}$-specific or *bla*$_{NDM-1}$-specific probe, respectively.

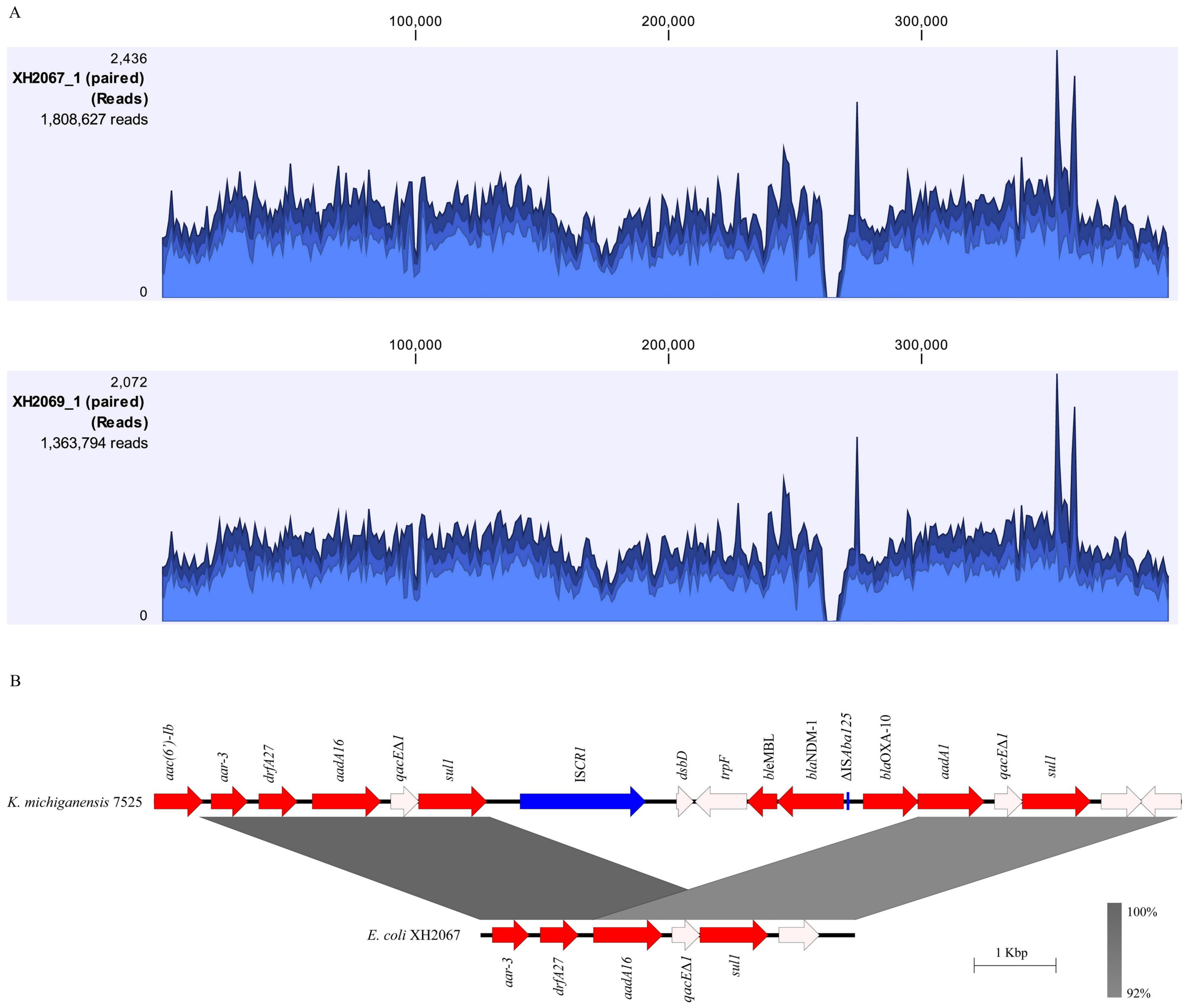

Supplementary Figure 2. Genome alignment between the *E. coli* transconjugants after experimental passage and *K. michiganensis* strain 7525 in the complete plasmid pKOX7525_1.

(A) Mapping graphs representing read coverage difference between the genome of XH2067 (or XH2069) and the reference sequence (pKOX7525_1). (B) Scaled, linear diagrams comparing the sequences between XH2057 and pKOX7525_1. Gray shading and squares in colors indicate homologies between the corresponding genetic loci on each plasmid. Arrows indicate open reading frames, with arrowheads indicating the direction of transcription: red, antibiotic-resistant encoding genes; blue, transposon- and integron-associated genes; Other genes are shown as white arrows.

Supplementary table 1. Strain list used in this study

Name	Description
Klebsiellia michiganensis strain 7525	Clinical strain studied in our work co-harboring *bla*$_{IMP-4}$ and *bla*$_{NDM-1}$ on the same plasmid, wild-type
E. coli J53	Lab stain *Escherichia coli* J53, recipient used for transconjugation experiment, sodium azide-resistant
E. coli J53 (pKOX7525_1)	Transconjugant, *E. coli* J53 containing pKOX7525_1
XH2065	Single colony being positive with both *bla*$_{IMP-4}$ and *bla*$_{NDM-1}$ gene, selected from population of *E. coli* J53 (pKOX7525_1) after ten days of passage in LB broth with 1μg/mL ciprofloxacin
XH2066	Single colony being positive with *bla*$_{IMP-4}$ gene but negative with *bla*$_{NDM-1}$, selected from population of *E. coli* J53 (pKOX7525_1) after ten days of passage in LB broth with 1μg/mL ciprofloxacin
XH2067	Single colony being positive with *bla*$_{IMP-4}$ gene but negative with *bla*$_{NDM-1}$, selected from population of *E. coli* J53 (pKOX7525_1) after ten days of passage in LB broth without antibiotic,
XH2068	Single colony being negative with both *bla*$_{IMP-4}$ and *bla*$_{NDM-1}$, selected from population of *E. coli* J53 (pKOX7525_1) after ten days of passage in LB broth without antibiotic
XH2069	Single colony being positive with *bla*$_{IMP-4}$ but negative with *bla*$_{NDM-1}$, selected from population of *E. coli* J53 (pKOX7525_1) after five days of passage in LB broth without antibiotic

Supplemental table 2. Genomic information and antimicrobial resistance gene of *K. michiganensis* strain 7525

Name	Size(bp)	G+C (%)	Antimicrobial resistance genes (copy number)	Accession number
K. michiganensis strain 7525 chromosome	5,847,354	55.69	bla_{OXY-5}, $bla_{CTX-M-15}$	CP065474
pKOX7525_1	397,447	49.10	bla_{OXA-16}, bla_{SFO-1}, bla_{IMP-4}, bla_{NDM-1}, *aac(6')-Ib-cr* (3 copies), *aac(6')-Ib3*, *aph(6)-Id*, *aph(3'')-Ib*, *qnrS1*, *qacE* (2), *aadA16*, *aadA1* (2), *sul1* (2), *mph(A)*, *catB3*, *ARR-3*, *dfrA27*	CP065475
Plasmid 2	6,397	44.35	ND	NA
Plasmid 3	3,662	46.56	ND	NA

NA, not applied. ND, not detected.

Supplementary table 3. Bioinformatic information of multiple carbapenemase producing CREs reported in China

Case.	Species (Number)	Isolate name	Plasmid name	Carbapenem-resistant gene	Size (bp)	Inc type	year	GenBank No.	Reference
1	*Klebsiella oxytoca* (1)	KOX3	pKOX3-P5-KPC	$bla_{\text{KPC-2}}$	40,277	IncP6	2017	KY913901	[1]
			pKOX3-P3-NDM	$bla_{\text{NDM-1}}$	55,246	IncX3		KY913899	
			pKOX3-P4-IMP	$bla_{\text{IMP-4}}$	61,680	IncN		KY913900	
2	*Providencia rettgeri* (1)	Strain 2055	p2055-IMP	$bla_{\text{NDM-1}}$, $bla_{\text{IMP-4}}$	41,936	IncW	2018	MH882484	None
3	*Enterobacter cloacae* (2)	SZECL1	pKPC3_SZ	$bla_{\text{KPC-3}}$	43,333	IncX6	2016	KU302800	[2]
			pNDM1_SZ1	$bla_{\text{NDM-1}}$	130,573	IncA/C2+IncR		KU302801	
		SZECL2	pKPC3_SZ	$bla_{\text{KPC-3}}$	43,333	IncX6		KU302800	
			pNDM1_SZ2	$bla_{\text{NDM-1}}$	132,232	IncA/C2		KU302802	
4	*Citrobacter freundii* (1)	Strain 112298	p112298-KPC	$bla_{\text{KPC-2}}$	117,288	No hit found	2015	KP987215	[3]
			p112298-NDM	$bla_{\text{NDM-1}}$	53,489	IncX3		KP987216	
6	*Klebsiella pneumoniae* (7)	C2315	pC2315-4-KPC	$bla_{\text{KPC-2}}$	175,540	IncFIA(HI1)+IncFII	2020	NZ_CP039831	[4]
			pC2315-2-NDM	$bla_{\text{NDM-1}}$	317,231	IncHI1B		NZ_CP039829	
		C2343	pC2343-4-KPC	$bla_{\text{KPC-2}}$	175,540	IncFIA(HI1)+IncFII		NZ_CP039826	
			pC2343-2-NDM	$bla_{\text{NDM-1}}$	318,848	IncHI1B		NZ_CP039824	
		C2414	pC2414-2-KPC	$bla_{\text{KPC-2}}$	172,001	IncFII(pHN7A8)+IncR		NZ_CP039820	
			pC2414-3-NDM	$bla_{\text{NDM-1}}$	63,046	IncN		NZ_CP039821	
		C2660	pC2660-3-KPC	$bla_{\text{KPC-2}}$	153,556	IncFII(pHN7A8)+IncR		NZ_CP039810	
			pC2660-4-NDM	$bla_{\text{NDM-1}}$	53,097	IncX3		NZ_CP039811	
		C2601	pC2601-3-KPC	$bla_{\text{KPC-2}}$	97,386	IncFII(Yp)		NZ_CP039815	
			pC2601-5-NDM	$bla_{\text{NDM-1}}$	51,995	IncN2		NZ_CP039817	
		C2972	pC2972-4-KPC	$bla_{\text{KPC-2}}$	97,386	IncFII (Yp)		NZ_CP039805	
			pC2972-5-NDM	$bla_{\text{NDM-1}}$	51,995	IncN2		NZ_CP039806	
		C2974	pC2974-4-KPC	$bla_{\text{KPC-2}}$	97,466	IncFII(Yp)		NZ_CP039798	
			pC2974-6-NDM	$bla_{\text{NDM-1}}$	51,995	IncN2		NZ_CP039800	

References

[1] J. Wang, M. Yuan, H. Chen, X. Chen, Y. Jia, X. Zhu, L. Bai, X. Bai, S. Fanning, J. Lu, and J. Li, First Report of Klebsiella oxytoca Strain Simultaneously Producing NDM-1, IMP-4, and KPC-2 Carbapenemases. Antimicrob Agents Chemother 61 (2017).

[2] H. Du, L. Chen, K.D. Chavda, R. Pandey, H. Zhang, X. Xie, Y.W. Tang, and B.N. Kreiswirth, Genomic Characterization of Enterobacter cloacae Isolates from China That Coproduce KPC-3 and NDM-1 Carbapenemases. Antimicrob Agents Chemother 60 (2016) 2519-23.

[3] J. Feng, Y. Qiu, Z. Yin, W. Chen, H. Yang, W. Yang, J. Wang, Y. Gao, and D. Zhou, Coexistence of a novel KPC-2-encoding MDR plasmid and an NDM-1-encoding pNDM-HN380-like plasmid in a clinical isolate of Citrobacter freundii. J Antimicrob Chemother 70 (2015) 2987-91.

[4] H. Gao, Y. Liu, R. Wang, Q. Wang, L. Jin, and H. Wang, The transferability and evolution of NDM-1 and KPC-2 co-producing Klebsiella pneumoniae from clinical settings. EBioMedicine 51 (2020) 102599.
